# Open-label study of efgartigimod in seronegative myasthenia gravis

**DOI:** 10.1177/17562864251388019

**Published:** 2025-10-31

**Authors:** Mohamed Khateb, Ajith Sivadasan, Carolina Barnett-Tapia, Lubna Daniyal, Lahiru Fernando, Shiyi Chen, Leif Erik Lovblom, Hans Katzberg, Vera Bril

**Affiliations:** Ellen and Martin Prosserman Centre for Neuromuscular Diseases, University Health Network, University of Toronto, Toronto, ON, Canada; Ellen and Martin Prosserman Centre for Neuromuscular Diseases, University Health Network, University of Toronto, Toronto, ON, Canada; Ellen and Martin Prosserman Centre for Neuromuscular Diseases, University Health Network, University of Toronto, Toronto, ON, Canada; Institute of Health Policy, Management and Evaluation, University of Toronto, Toronto, ON, Canada; Ellen and Martin Prosserman Centre for Neuromuscular Diseases, University Health Network, University of Toronto, Toronto, ON, Canada; Ellen and Martin Prosserman Centre for Neuromuscular Diseases, University Health Network, University of Toronto, Toronto, ON, Canada; Biostatistics Department, University Health Network, Toronto, ON, Canada; Institute of Health Policy, Management and Evaluation, University of Toronto, Toronto, ON, Canada; Biostatistics Department, University Health Network, Toronto, ON, Canada; Ellen and Martin Prosserman Centre for Neuromuscular Diseases, University Health Network, University of Toronto, Toronto, ON, Canada; Ellen and Martin Prosserman Centre for Neuromuscular Diseases, University Health Network, 5EC-309, Toronto General Hospital, University of Toronto, 200 Elizabeth Street, Toronto, ON M5G 2C4, Canada

**Keywords:** efgartigimod, myasthenia gravis, seronegative, study, treatment

## Abstract

**Background:**

The ADAPT trial demonstrated the benefit of efgartigimod, a neonatal Fc receptor (FcRn) inhibitor, in acetylcholine receptor antibody (AChRAb) positive patients with generalized myasthenia gravis (MG). Information regarding the benefits in those lacking pathogenic antibodies is sparse.

**Objectives:**

We aimed to investigate the safety and efficacy of efgartigimod in patients with double-seronegative (SN) generalized MG.

**Design:**

An open-label 6-month prospective study, conducted at our center.

**Methods:**

Patients aged at least 18 years with clinical and electrodiagnostic features of MG and negative results for AChRAb and muscle-specific tyrosine kinase antibodies were included. Efgartigimod was administered weekly for 4 weeks and then biweekly for 5 months followed by an observation period. The primary endpoint was the change in MG impairment index (MGII) at 6 months compared to baseline. Secondary endpoints include the change in MG activities of daily living (MG-ADL), other MG scores, overall responders, and early responders. The safety analysis included all patients who received at least one dose of efgartigimod.

**Results:**

We enrolled 30 patients with SN MG who were resistant to other treatments and had unacceptable MGII scores. The MGII decreased by 11.92 points (*p* < 0.01) with efgartigimod treatment. The MG-ADL also improved. Seventy-two percent of patients were responders with 31% being early responders. Adverse events were reported in 83.3% of patients, and in 90.6%, they were mild. Headache was the most common, reported in 26.7%, followed by flu/common cold in 20%, and urinary tract infection in 13.3%.

**Conclusion:**

Efgartigimod was well tolerated and efficacious in patients with SN MG. Future randomized, placebo-controlled studies are needed.

**Trial registration:**

This trial is registered at ClinicalTrials.gov (NCT 06587867), accessed via https://clinicaltrials.gov/study/NCT06587867?locStr=Toronto,%20ON,%20Canada&country=Canada&state=Ontario&city=Toronto&cond=Myasthenia%20Gravis&intr=efgartigimod&rank=1

## Plain language summary

The safety and efficacy of the novel treatment efgartigimod in myasthenia gravis with negative antibodies

We enrolled 30 patients with myasthenia gravis with negative antibodies. All patients received the new treatment Efgartigimod and we compared the severity of their myasthenia symptoms with the treatment, compared to the baseline (before giving it). The severity of the myasthenia was significantly reduced (observed in multiple clinical scores for myasthenia gravis). Adverse events were reported in 83.3% of patients, and in 90.6% they were mild. Headache was the most common, reported in 26.7%, followed by flu/common cold in 20%, and urinary symptoms in 13.3%. To conclude, in this study, we showed, for the first time that the novel treatment of Efgartigimod is well tolerated and efficacious in patients with Myasthenia gravis with no antibodies. Future prospective and placebo-controlled studies are needed.

## Introduction

Myasthenia gravis (MG) is an autoimmune disease, characterized by muscle weakness and fatigability of skeletal muscles. In most MG patients (~85%), antibodies against the acetylcholine receptor (AChR) are detected.^1,2^ However, no antibodies against the AChR are detected in about 15% of patients with generalized MG and up to 50% of patients with ocular forms of the disease.^3,4^ Furthermore, antibodies against muscle-specific tyrosine kinase (MuSK) are found in approximately 6% of the patients, while antibodies against low-density lipoprotein receptor-related protein 4 (LRP4) are present in about 2% of MG patients.^5,6^ MG cases without these antibodies are identified as “double or triple seronegative (SN) MG.” Literature regarding the efficacy of classical therapies in double SN MG is sparse compared to seropositive MG. SN MG is associated with a higher risk of treatment failure.^7,8^ In addition, refractory SN MG patients have worse Myasthenia gravis Foundation of America (MGFA) classes compared with AChR antibody (AChRAb) seropositive refractory patients implying a more aggressive disorder.^9^ SN MG patients may represent a distinct group with unmet therapeutic needs who might require more individualized and targeted treatment approaches.

There have been recent advances with novel therapies for MG such as B cell depletion therapies, complement inhibitors, and neonatal Fc receptor antagonists, especially in antibody-positive MG patients.^10–13^ Unfortunately, none of these novel treatments were tested specifically in SN MG other than nipocalimab in a trial done recently. One candidate strategy is based on inhibition of the neonatal crystallizable fragment (Fc) neonatal receptor (FcRn). FcRn was shown to play a central role in trafficking IgGs and albumin into recycling pathways rescuing them from lysosomal degradation.^14,15^ Efgartigimod (ARGX-113) is a human IgG1-derived Fc fragment of the za allotype that binds to human FcRn resulting in blockage of FcRn-mediated recycling of IgGs.^12,13^ By inhibiting this FcRn function, efgartigimod leads to rapid degradation of IgGs, which are expected to modify IgG-driven autoimmune diseases. In addition, efgartigimod selectively reduced IgG and does not lead to reduction in albumin or cholesterol.

Evidence from preclinical studies supported the concept of inhibiting FcRn in IgG-driven autoimmune disorders.^16,17^ Subsequent randomized, placebo-controlled phase II and phase III trials in generalized MG have shown efgartigimod to be effective as well as safe.^18,19^ In the pivotal ADAPT trial, efgartigimod was associated with a favorable clinical response compared to placebo leading to regulatory approval for use in generalized AChRAb positive MG with a good safety profile. Efgartigimod was administered in a dose of 10 mg/kg once weekly for 4 weeks followed by on demand therapy if clinical deterioration occurred and this regimen produced significant clinical benefits.^19^ In the ADAPT NXT study, efgartigimod was administered in a dose of 10 mg/kg weekly for 4 weeks followed by treatment once every 2 weeks and showed the long-lasting benefits of this treatment. Regarding safety, efgartigimod was well tolerated with similar safety profiles regardless of dosing regimen in IgG-mediated autoimmune diseases.^20,21^ Most common adverse effects were headache (29%), followed by upper respiratory tract infection (URTI)/nasopharyngitis (12%), and urinary tract infection (UTI) (10%), with only 5% of all the reported side effects considered as serious.^19^

In the pivotal ADAPT study, a similar 68% response rate was observed in SN MG patients as in AChRAb positive patients based on MG activities of daily living (MG-ADL) scale, but a high placebo response rate (63%) meant the results were inconclusive in these patients leading to a need for additional study in SN MG patients. Specifically, a post hoc analysis for the 32 double seronegative patients showed a 48% responder rate, in both MG-ADL and quantitative myasthenia gravis (QMG), in the efgartigimod group, compared to 21% in placebo. A more intense treatment regimen is predicted to achieve a response in SN MG as studies have shown lesser responsiveness in these patients.^7,8^

We aimed to investigate the efficacy and safety of the novel biological treatment, efgartigimod, in SN MG patients.

## Methods

### Study design

We conducted an open-label 6-month prospective study of efgartigimod treatment in patients with AChRAb and MuSK antibody negative MG from June 2023 to March 2025 with moderate to high severity SN MG attending our tertiary medical facility. The primary objective was to evaluate the long-term efficacy, safety, and tolerability of efgartigimod in these patients. This study was approved by the local University Health Network research ethics committee, and followed the principles outlined in the Declaration of Helsinki. Patients provided written informed consent. The date of ethical approval was May 23, 2023.

### Participant selection

All male and female patients aged 18 years or more, with SN MG for AChR and MUSK antibodies, and with MGFA classes of II–IV, were eligible for screening. The gold standard for a SN MG diagnosis in our study was the combination of both the clinical features of MG and an abnormal electrodiagnostic (EDX) test (single-fiber EMG [SFEMG] or repetitive nerve stimulation studies [RNS]) with negative serology for AChR and MUSK antibodies using radioimmunoprecipitation assays. MG severity was evaluated according to the Myasthenia Gravis Impairment Index (MGII), MG-ADL score, patient-acceptable symptom state (PASS) response, and the single simple question (SSQ). Cell-based antibody assays and genetic testing for congenital MG were not done.

At screening, potential participants were required to have MGII score >11 or MG-ADL score of at least 5 (with >50% of the score due to nonocular items), a PASS response of “No” and an SSQ of <70% with at least 6 months of historical data at the baseline. Patients were required to be on stable doses of their standard of care (SoC) MG treatment for at least 1 month prior to screening. Patients were enrolled if their symptoms/MGII values were stable or worsening during the run-in period. The SoC included acetylcholinesterase inhibitors, steroids, and nonsteroidal immunosuppressants (NSIST) such as azathioprine, methotrexate, cyclosporine, tacrolimus, and mycophenolate mofetil.

**Figure 1.**
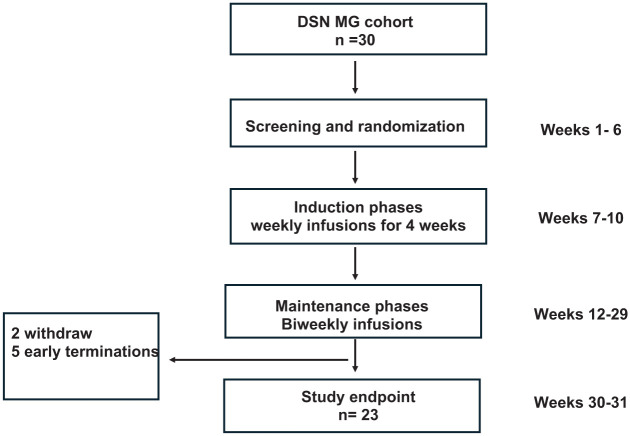
Trial profile. A flow chart summarizing the trial, including the number of patients in each phase, withdrawals, and early terminations.

Patients were excluded if they had received rituximab or eculizumab in the 6 months before screening, if they had thymectomy during the last 3 months before screening, or had intravenous immunoglobulin or plasma exchange within 4 weeks of screening. Patients having autoimmune disorders other than MG were excluded. Seropositivity for hepatitis B virus, hepatitis C virus, or human immunodeficiency virus, or clinically significant uncontrolled active or chronic bacterial, viral, or fungal infection at screening resulted in the patient’s exclusion. Additional exclusion criteria included recent major surgery, which could confound the results of the trial or put the patient at undue risk. Patients with renal or hepatic dysfunction defined by creatinine >1.5× and/or transaminases >2.5× the upper limits of normal at screening were excluded. The flow of patients is shown in Figure 1.

### Treatment and investigational product

**Figure 2.**
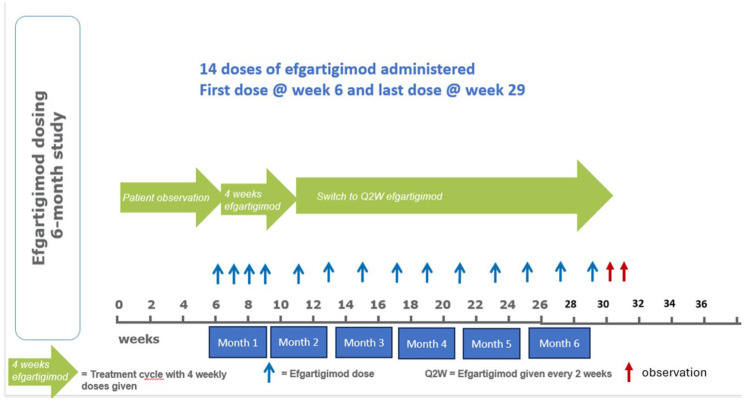
The detailed study design emphasizing the screening phase, patient observation phases, induction and maintenance phases, infusion visits (blue arrows), observation visits (red arrows), and study endpoint.

In this trial, the study drug was efgartigimod alpha (ARGX-113), which is a human anti-FcRn IgG1 Fc fragment. First, patients were observed for a 6 week run-in period and then efgartigimod was administrated at 10 mg/kg IV once weekly for 4 weeks (“induction therapy”), followed by 10 mg/kg IV once every 2 weeks (“maintenance phase therapy”) until the end of the study at 6 months (Figure 2). The IV infusion took 1 h in the outpatient clinic. The total dose per efgartigimod infusion was capped at 1200 mg for patients with body weight ⩾120 kg.

We collected MGII, SSQ, and PASS data from the 12 months preceding study entry and these data plus other end-points (MG-ADL, QMGS, etc.) from the 6 week run-in period to use as baseline data. Patients were enrolled if their status was stable or worsening during the run-in period. In this context, stable does not equate to being well. At the end of the study, the change in each of the scores and the percentage of responders to the study drug were determined.

### Study endpoints

The primary outcome was the change from baseline in MGII score at 6 months. MGII has 22 patient-reported and 6 physician-assessed items for a composite score ranging from 0 to 84, with a higher score signifying greater disability. The MGII has less floor effect than the MG-ADL and is simpler, less time-consuming, and centered mainly around the patient’s symptoms compared to physical examination in the QMGS. It has a greater utility in assessing the severity in the ocular domains as well as fatigue. The individual patient minimum important difference (MID) for the MGII score is 5.5.^22^ A patient is considered a “responder” if there is an improvement of ⩾5.5 points in the MGII compared to baseline.^22^ This cutoff should produce an estimation of MGII responders with a 64% sensitivity and 73% specificity. We chose to round this threshold to 6 points to prevent interrater or intervisit variability and confusion when trying to label individual patients as responders or not. The MGII correlates strongly with other patient-related outcome scores, such as the PASS and SSQ.^23,24^ The PASS response is a “Yes” or “No” answer about whether a patient is satisfied with their current MG status and provides a holistic overview of their MG state.^24^ The SSQ is a simple and validated question of what percentage of normal a patient feels concerning their MG, with 100% being normal.^25^

Secondary outcomes included changes in MG-ADL score, QMG score, Myasthenia gravis quality of life-revised (MG-QOLr) score, PASS response, SSQ, and the percentage of early and overall responders to the study drug. Early responders were defined as patients who responded by at least a 6 point reduction in MGII, or at least 2 points in ADL, within 4 weeks of infusion.

### Safety assessments

Safety outcome was achieved by evaluating the incidence and severity of adverse events (AEs), serious adverse events, severe adverse events, vital signs, and laboratory assessments throughout the entire duration of the trial. All AEs observed were graded using the National Cancer Institute Common Terminology Criteria for Adverse Events version 5.0.dfg.

### Statistical analysis

A power analysis was conducted to determine the sample size required for this study, which was 30. This was based on a previously published study about MGII responsiveness.^22^

Descriptive statistics included counts and proportions for categorical variables, and means (standard deviations) and medians (interquartile ranges, IQR) for continuous variables. Box plots were generated to visualize the patterns of MGII, MG-ADL, QMG, MG-QOLr scores from screening to study termination. The difference in scores between the first infusion and various follow-up time points was also calculated. Box plots were used to assess the patterns of the differences scores across time. Bar charts were plotted to examine the proportion of patients with PASS = Yes at various time points. Similarly, the difference in proportions between the first infusion and various follow-up time points was obtained and plotted. Pearson’s correlations were used to determine the associations between the continuous scores. Two sample *t* tests were used to examine the differences in scores between PASS = Yes and PASS = No groups.

Univariable linear mixed models were used to examine changes in MGII, MG-ADL, QMG, and MG-QOLr over time. Multivariable linear mixed models were also built to adjust for the effects of confounders such as age, disease duration, and comorbidities including cardiovascular disease, respiratory disease, and thymectomy. The correlations between repeated measures within the same patients were accounted for in these models.

Univariable generalized estimating equation models were built to assess the effects of efgartigimod on the dichotomous secondary outcome PASS, while accounting for the correlations amongst longitudinal repeated measures.

All statistical analyses were carried out using SAS 9.4 software (Cary, NC, USA). Two-sided tests were used, and the statistical significance level was set at *p* < 0.05.

## Results

### Patient characteristics, electrodiagnostic, and clinical features

Of about 100 double SN MG patients followed in our clinic between 2022 and 2025, 30 patients met the inclusion and exclusion criteria and agreed to participate in the study. The majority were considered treatment-resistant before the trial, meaning the persistence of significant MG impairments after trials of steroids, NSIST, and IVIG or PLEX.

Table 1 shows the demographics of this study cohort. The median age of patients was 62.5 (IQR = 55–69) years at screening, with males constituting 26.67% (8/30) of the cohort. At screening, the mean MGII was 44.6 ± 5.1, and the mean MG-ADL was 10.6 ± 1.44. The mean jitter on SFEMG was 84.3 (IQR = 37.7–234) µs, and the percentage of abnormal pairs was 41% (14%–79%). Pathology results following thymectomy revealed normal thymic tissue in 54.5%, hyperplasisa in 27.27%, and thymoma in 18.18%. The median time from thymectomy to screening was 9 (IQR = 0.5–17) years. The leading medical comorbidity was hypertension in 53.3% of patients. Notably, heart and respiratory conditions were identified in 30% and 10%, respectively. In 33.33%, a combination of two different immunosuppressants was used at screening. The leading combination was prednisone and mycophenolate in 20%. On average, the number of previously failed immunosuppressant and immunomodulatory treatments for the cohort was 2 (range 0–6).

**Table 1.** Demographics and basic features.

Variable	Cohort (*n* = 30)
Median age (years) (IQR)	62.5 (55.0–69.0)
Males (%, *n*)	26.7 (8/30)
Duration of disease (years)—median (IQR)	8.0 (3.0–12.0)
Disease course (in the last year prior to screening)	Satisfactory—stable: 10% (3/30) Satisfactory—unstable: 10% (3/30) Unsatisfactory—stable: 36.67% (11/30) Unsatisfactory—unstable: 43.33% (13/30)
MGII at screening (average)	44.6 ± 5.1
MG-ADL at screening (average)	10.6 ± 1.44
Ratio of “Yes” PASS at screening	0
SSQ	44.6 ± 18.7
Abnormal SFEMG ratio (number) (%, *n*)	100 (30/30)
Abnormal RNS ratio (number) (%, *n*)	14.8 (4/27)
Thymectomy (%, *n*)	36.7 (11/30)
Thymoma (%, *n*)	18.2 (2/11)
Hyperplasia (%, *n*)	27.3 (3/11)
Normal thymus (%, *n*)	54.5 (6/11)
Comorbidities (%, *n*)	
Hypertension	53.3 (16/30)
Major surgeries in the past	40 (12/30)
Heart failure and other cardiac issues	30 (9/30)
Anxiety and/or depression	30 (9/30)
Diabetes	23.3 (7/30)
Dyslipidemia	20 (6/30)
Osteoarthritis	20 (6/30)
Spinal structural pathologies	20 (6/30)
Tumors (including thymoma)	16.7 (5/30)
Anemia	13.3 (4/30)
Respiratory disorders	10 (3/30)
Pancreatitis	10 (3/30)
Cerebrovascular disease	6.7 (2/30)
Hemato proliferative disorders	6.7 (2/30)
Necrotizing fasciitis	3.3 (1/30)
Spinal cord compression	3.3 (1/30)
Pulmonary embolism	3.3 (1/30)
Sarcoidosis	3.3 (1/30)
Peripheral vascular disease	3.3 (1/30)
Chronic kidney disease	3.3 (1/30)
Active medications at enrollment	
Mestinon (%, *n*)	70 (21/30)
Prednisone (%, *n*)	70 (21/30)
Mycophenolate (%, *n*)	36.7 (11/30)
Azathioprine (%, *n*)	10 (3/30)
Methotrexate (%, *n*)	6.7 (2/30)
Tacrolimus (%, *n*)	3.3 (1/30)
Plasmapheresis (%, *n*)	3.3 (1/30)
Chronic IVIG (%, *n*)	0
SCIGs (%, *n*)	0
Cyclosporine (%, *n*)	0
Cyclophosphamide (%, *n*)	0
Rituximab (%, *n*)	0
Active treatment combinations at enrollment	
Prednisone and mycophenolate (%, *n*)	20 (6/30)
Prednisone and azathioprine (%, *n*)	6.7 (2/30)
Prednisone and methotrexate (%, *n*)	3.3 (1/30)
Prednisone and tacrolimus (%, *n*)	3.3 (1/30)
Further details about the baseline doses and duration of treatment doses before screening	
Baseline prednisone dosage (mg/day)	14.3 (5–30)
Duration on prednisone screening dose (from onset, in months)	43.1 (1.5–219)
Baseline mycophenolate dosage (mg/day)	1864 (720–3000)
Duration on mycophenolate screening dose (from onset, in months)	32.7 (3–102)
Baseline azathioprine dosage (mg/day)	125 (100–175)
Duration on azathioprine screening dose (from onset, in months)	16.3 (9–28)

Data presented as mean ± SD, median (IQR), or percentage (count/30).

IQR, interquartile range; MG-ADL, myasthenia gravis activities of daily living; MGII, myasthenia gravis impairment index; PASS, patient-acceptable symptom state; SCIG, subcutaneous immunoglobulins; SSQ, single simple question.

### The effect of efgartigimod on MGII and other scores at study endpoint

**Figure 3.**
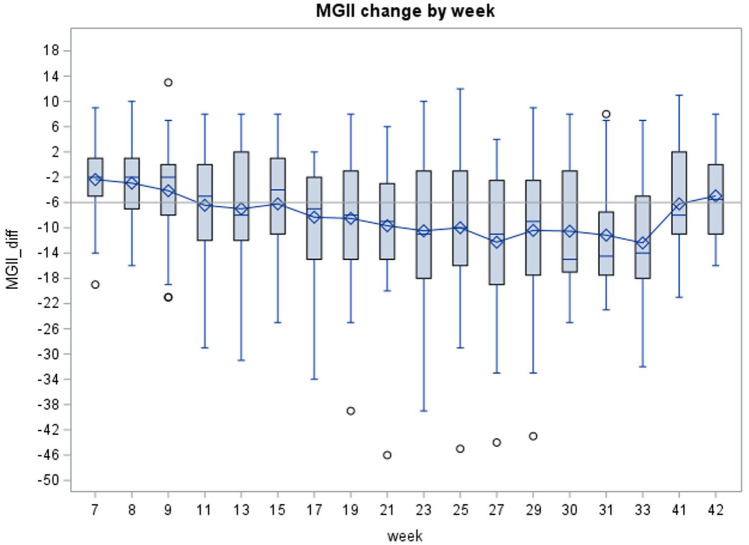
Change of MGII with time. The *x*-axis represents time in weeks at the induction and maintenance phases (starting from week 7), and at the study endpoint. Error bars show standard error. The horizontal line indicates the minimal clinically important difference for the MGII (6 points). The horizontal hatched line indicates zero change. Circles represent outlier points. MGII, myasthenia gravis impairment index.

The MGII was reduced by a mean of 11.92 (95% CI = 5.19–18.65) points at weeks 30–31, and by 9.7 (95% CI = 26.9–38.6) points at week 29 (last infusion date), compared to baseline. Specifically, MGII was reduced from 44.6 ± 5.1 at baseline to 32.7 ± 5.9 at study endpoint (Table 2; Figure 3). This reduction was significant (*p* < 0.0008) before and after adjusting for covariates including age, duration of MG, cardiac disease, respiratory disease, and thymectomy (Table 1).

**Table 2.** Primary and secondary outcomes.

Score	Baseline	Endpoint	Change from baseline	*p*-Value
MGII	44.6 ± 5.1	32.7 ± 5.9	11.9 (5.19–18.65)	<0.001
MG-ADL	10.6 ± 1.44	7.2 ± 1.6	3.4 (1.72–5.07)	<0.001
QMG	16.6 ± 2.09	15.9 ± 2.38	0.7 (−1.86 to 3.35)	0.57
MG-QOLr	18.05 ± 2.94	14.9 ± 3.19	3.2 (0.25–6.05)	0.034
PASS yes (%)	0	24	24	0.048
SSQ	44.6 ± 18.7	56.9 ± 23.2	12.4	0.043

MG-ADL, myasthenia gravis activities of daily living; MGII, myasthenia gravis impairment index; MG-QOLr, myasthenia gravis quality of life-revised; PASS, patient-acceptable symptom state; QMG, quantitative myasthenia gravis; SSQ, single simple question.

**Figure 4.**
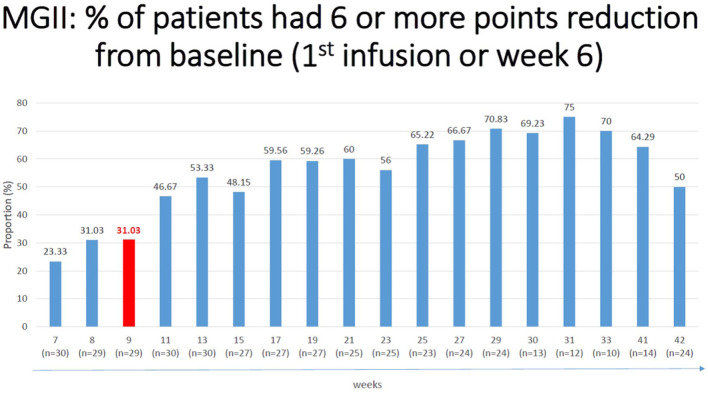
Percentage of patients with clinically meaningful reduction in MGII compared to baseline. MGII, myasthenia gravis impairment index.

**Figure 5.**
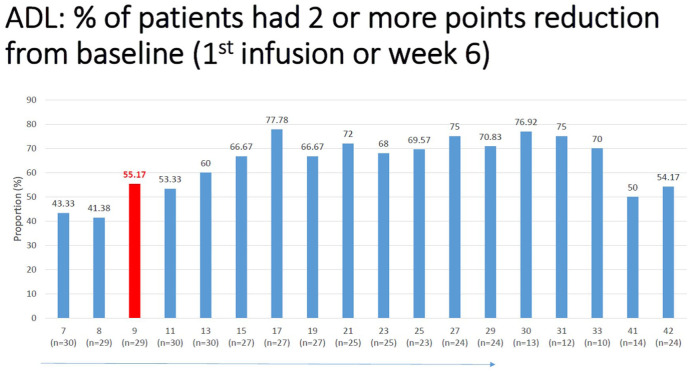
Percentage of patients with clinically meaningful reduction in MG-ADL compared to baseline. MG-ADL, myasthenia gravis activities of daily living.

Similarly, MG-ADL was improved by 2.7 points at weeks 29 and by 3.4 points at weeks 30–31, compared to baseline (*p* < 0.05; Table 2). Comparing QoL and PASS “yes” ratio scores also improved as shown in Table 2. The QMG score showed no significant change at study endpoint. However, in 33.33% (10/30) of the patients, a reduction of 3 or more points (which is considered the minimally clinically important difference or MCID) was observed in QMG. At study endpoint, 72% and 76% of patients met the criteria for “responder,” defined as achieving a 6-point reduction in MGII or 2-point drop in their MG-ADL (Figures 4 and 5). Of the 30 patients, none became symptom-free.

A “Last Observation Carried Forward” sensitivity analysis was performed to address the issue that the first 10 patients in the study lacked observation time points at weeks 30 and 31. Therefore, the study endpoint for these 10 patients was carried forward from week 29. The results were similar showing a mean 11.4 point reduction in MGII between study endpoint and baseline (*p* = 0.001), and a 3.0 point reduction in MG-ADL (*p* < 0.0001). MG-QOLr decreased by 3.2 points at study endpoint compared to baseline, *p* = 0.005. QMG and PASS did not change significantly, but by week 31, 24% reported PASS yes.

The following clinical and electrophysiologic parameters were explored as potential biomarkers predicting a positive response to efgartigimod: disease duration, thymectomy, number of failed medications in the past, baseline MGII, baseline ADL, average jitter, ratio of abnormal pairs. For each of these parameters, we compared the “responder” and the “nonresponder” subgroups. None predicted response to treatment, although the baseline MGII showed a trend, with higher MGII values (50.3 ± 13.0) in responders compared with 38.6 ± 4.3 in nonresponders, *p* = 0.059.

### Dynamics of response to efgartigimod during treatment cycles

Early responders were defined as patients who responded by at least a 6 point reduction in MGII, or at least 2 points in ADL, within 4 weeks of the first infusion. More than half the patients (55%) successfully met this criterion in ADL, and almost a third (31%) in MGII (Table 3). The responders increased with treatment time, reaching 72% for MGII and 76% for ADL scores (Figures 4 and 5).

**Table 3.** Responder analysis.

Category	MGII	MG-ADL
Early responders	31	55
Late responders	41	21
Overall responders	72	76

Values are percentages.

MG-ADL, myasthenia gravis activities of daily living; MGII, myasthenia gravis impairment index.

A similar effect was observed for the PASS parameter, as 0% of patients had a “yes” response to PASS at baseline. The ratio of patients with “yes” PASS increased to 24% at the study endpoint, and decreased to 8.3% at week 42, the end of the observation period.

### Safety outcome

AEs were reported in 83.3% of patients (Table 4). Headache was the most common, reported in 26.7% of patients, followed by flu/common cold in 20%, and urinary tract infection in 13.3%. Additional AEs included gastrointestinal symptoms of nausea and diarrhea, shingles, COVID19 infection, URTI, gastroenteritis, and fatigue (Table 4). Most AEs were mild (90.6%), while the remaining 9.4% were moderate. No severe AEs were identified. Of the moderate AEs, meningitis, bacteremia, and parapneumonic effusion were reported in the same patient causing a hospital admission and a delay of 3 months in infusion number 8. This event was considered unrelated to treatment. Treatment had to be delayed in 10% (3/30), twice in two of them. The delays were due to COVID 19 infection in two patients, meningitis infection, a fall leading to hospital admission, and MG worsening. No mortality was recorded during the study.

**Table 4.** Safety and AEs in 30 Patients.

Adverse Event	Percentage (number/30)
Any AEs	83.3 (25/30)
Any serious AEs	0
Any AEs leading to the discontinuation of the study drug	0
Bacterial infections	13.3 (4/30)
Viral infections	20 (6/30)
Infusion-related reactions	0
Most common related AEs	
Headaches	26.7 (8/30)
Flu/influenzas	20 (6/30)
UTI	13.3 (4/30)
Nausea	10 (3/30)
Diarrhea	10 (3/30)
COVID19	10 (3/30)
Gastroenteritis	10 (3/30)
Shingles	6.7 (2/30)
URTI	3.3 (1/30)
Fatigue	3.3 (1/30)
Meningitis bacteremia	3.3 (1/30)
Parapneumonic effusion	3.3 (1/30)

Values are percentages.

AE, adverse event.

## Discussion

This prospective, open-label study in double SN MG patients showed that efgartigimod treatment reduces myasthenic impairments as measured by the MGII. Our results, which showed a significant reduction in the MGII at the study endpoint compared to baseline, achieved the primary outcome of the study. Improvements were observed in other MG scores, including MG-ADL, MG-QOLr, SSQ, and PASS, strengthening the results.

Double SN MG can be challenging to diagnose and to treat. It is reported to be associated with a higher risk of treatment failure,^7,8^ and the refractory form more aggressive, compared with AChRAb positive MG.^9^ In a previous study at our center, SN MG was found to be similar to AChRAb positive MG with respect to severity at presentation and response to immunosuppressants. However, the MGII improvement in the AChRAb positive cohort was significantly better than the SN group.^7^ While these results support an immune pathophysiology in many SN MG patients, they also indicate more resistance to treatment in these patients. The results of the current study show clear improvement in SN MG patients in the MGII with a mean improvement of 11.92 points which is above the MID estimate at the group level (8.1 points) with efgartigimod treatment.^22^ A large proportion of patients (72%) met the criterion of “responder” in MGII, at the study endpoint. Similar results were observed with other MG scores such as the MG-ADL, which demonstrated a drop by 3.39 points and with a responder rate of 76%. Interestingly, one-fourth of patients expressed a “yes” PASS following the treatment, compared to zero at baseline. This shows that there is still room for improvement in most patients. Despite SN MG patients having more severe disease in our study (baseline MG-ADL 10.6) compared to those in the ADAPT trial (baseline MG-ADL 9.2), the proportion of responders was similar in both studies. Specifically, responders based on MG-ADL changes were found in 68% of the ADAPT AChRAb positive cohort and 76% of our current SN MG cohort. Moreover, the proportion of the “early responders,” defined as patients who had a reduction of at least 2 MG-ADL points within 4 weeks of first infusion, was very similar to the ADAPT trial as well (55% in our cohort, compared to 57% in the ADAPT trial). The difference in baseline severity might explain why all but one of our patients failed to reach a “minimal manifestation” status despite having a clear response to efgartigimod. The minimum paired MGII and MG-ADL scores were 7 and 3, respectively, compared to baseline scores of 16 and 5 in this patient. In all the rest of the patients, paired MGII and MG-ADL scores did not drop below 14 and 5, respectively, with more than a third of the cohort still having an MGII above 30 even after the improvement from treatment. This implies a high level of resistance to treatment in our cohort. Despite this limitation, a clear response in MGII and most of the other MG scores was observed, supporting the immune basis of the disease and, more importantly, the promising role of the drug in treating SN MG patients. Notably, efgartigimod’s positive effect was not clearly observed in the QMG score. One possible explanation relates to the difference between the QMG score and the MGII. While the former was developed based on expert-defined limits of normal, the latter has reference ranges validated in previous trials.^25,26^ An additional crucial difference between the two is the lack of patient-reported data in the QMG, which may limit its responsiveness to show change. The exact contribution of patient-reported versus objective physician-reported data in our study results needs to be addressed in future studies. Finally, the changes in the other scales were above the placebo response rate in prior research trials^11,19^ raising doubts that a placebo effect alone could account for the changes observed in this study. Additional reasons include interrater variability, differences in evaluation timing relative to treatment cycles, and more. Nevertheless, a nonnegligible 33.33% (10/30) of patients achieved a reduction of 3 or more points in QMG (the MCID), which is considered the MCID. Moreover, QMG was found to be significantly correlated with MG-ADL and MGII, despite the lack of significance of its reduction. Specifically, the Pearson’s correlation between MG-ADL and QMG was equal to 0.63 with *p* < 0.0001. Similar results of positive correlation were found between the MGII and QMG; 0.748 Pearson’s correlation coefficient and *p* < 0.0001. Considering our poorly controlled cohort at baseline, the ratio of minimal clinical improvement and the positive correlation with other MG scores are consistent with the general improvement of the patients.

Regarding safety, efgartigimod was highly tolerable. It was mostly associated with mild AEs. AEs were reported in 83.3% (25/30) of our cohort, compared to 77% and 84% in the drug and placebo groups of the ADAPT trial, respectively. Headache, flu/common cold symptoms, urinary tract infection, and gastrointestinal symptoms were the most common reported AEs, similar to what was reported in the ADAPT study. One patient had a more severe clinical course with meningitis bacteremia and parapneumonic effusion, leading to a delay of 3 months in one of the infusions. This AE was not thought to be directly linked to the efgartigimod treatment as the patient was complicated with multiple comorbidities including ischemic heart disease, arrhythmia, poorly controlled type 2 diabetes, and more. In addition, the patient was initially admitted to the hospital due to confusion and parapneumonic effusion, and only later (during hospital stay) was the patient diagnosed with bacteremia.

The mechanism of action for efgartigimod in DSN MG is obscure. First, it is difficult to properly address the speculated mechanism of efgartigmod in seronegative MG because we do not truly know the patho-mechanism of the seronegative MG itself. If the disorder is mediated via unrecognized pathogenetic IgG antibodies, then efgartigimod might affect through enhancing their clearance process and inhibiting their recycling. In general, beside laboratory errors (manifested in “false negative” results for the antibodies) and immunosenescence process, a possible patho-mechanism for DSN MG is antibodies against unknown neuromuscular junction targets. In this situation, efgartigimod is expected to affect via inhibiting the recycling of such pathogenetic antibodies, similar to its effect in a Ach R Ab positive MG.

Our study has several limitations. First is the lack of a placebo-control group. As noted in the ADAPT trial, a remarkable 30% of MG patients showed a significant response to the placebo treatment. However, the high similarity, in the ratio of overall responders and the early responders, between our cohort and the efgartigimod group of the ADAPT trial supports a true treatment effect rather than simply a placebo response. Also, the degree of MG-ADL change in our cohort exceeded the placebo change in the ADAPT patients despite our cohort being more difficult to treat successfully. The significant positive correlations between the different MG scores also supports a real, rather than a placebo-related effect. Second, the single tertiary center nature of the cohort likely resulted in a bias toward more difficult cases, while milder cases might have continued follow-up at other centers. This bias might explain why none of our patients reached a sustained “minimal manifestation” status despite the clear response to efgartigimod. Third, another bias in our study was inclusion of treatment-resistant SN MG patients of the total cohort of SN MG patients followed at our center. This cohort consists of more difficult patients than the previously published DSN MG cohort by our group in 2023.^7^ Baseline MGII is 44.6 ± 5.1 compared to 21.3 ± 13.3 in our previous DSN MG cohort. Most of the patients were uncontrolled with previous treatments (median of two failed treatments), while the controlled SN MG patients were not included as the preference was to avoid any change in their treatment regimen that might cause clinical deterioration. Compared to our DSN MG cohort published in August 2023,^7^ the average change in MGII is more prominent in our current cohort (11.92 decrement vs 7.7 decrement in the previous one). Responders’ ratio now is higher as well (72% vs 59.8%). SSQ increased by 12.4% now compared to 8.7% in the previous cohort. However, none of the patients in the previous cohort received efgartigimod; thus, such a comparison is very limited. Additionally, the differences in the dosing protocol between our study and the ADAPT trial might limit our conclusions, as we applied an induction phase followed by fixed biweekly maintenance dosing while the ADAPT study had a pulsed, response-driven regimen. The dosing regimen in our study might be associated with less wearing-off or worsening compared to the ADAPT design. The relatively small number of patients might be a limitation. An additional limitation is the lack of consistent use of cell-based assays to determine the patient’s serological status, as most of the serological tests were done with radioimmunoprecipitation assays. The lack of testing for anti LRP4 antibodies is theoretical limitation; however, due to the very low incidence of this subtype (about a third of the MUSK incidence), we do not think this might have affected our results. It is also unknown how LRP4 patients respond to any therapy. Finally, the lack of a biomarker for SN MG may lead to misclassification of some patients. Despite these limitations, our study clearly shows positive effects of efgartigimod in SN MG patients with a prominent proportion of responders.

To conclude, this prospective, open-label study investigated the role of efgartigimod in treating SN MG patients. The results indicate that the novel selective IgG reduction mechanism by blocking the FcRn receptor with efgartigimod is well tolerated and effective in SN MG patients.

## Declarations

**Ethics approval and consent to participate:** This study was approved by the local University Health Network research ethics committee, and followed the principles outlined in the Declaration of Helsinki. Ethics approval number is 22-5968. Patients provided written informed consent to participate in the study.

**Consent for publication:** Not applicable.

**Availability of data and materials:** The data that support the findings of this study are available from the corresponding author on reasonable request for authorized investigators.
